# Reward-Processing Behavior in Depressed Participants Relative to Healthy Volunteers: A Systematic Review and Meta-analysis

**DOI:** 10.1001/jamapsychiatry.2020.2139

**Published:** 2020-07-29

**Authors:** D. Chamith Halahakoon, Karel Kieslich, Ciarán O’Driscoll, Akshay Nair, Glyn Lewis, Jonathan P. Roiser

**Affiliations:** 1 Institute of Cognitive Neuroscience, University College London, London, England; 2 Department of Psychiatry, University of Oxford, Oxford, England; 3 Division of Psychiatry, University College London, London, England; 4 Max Planck Centre for Computational Psychiatry and Aging Research, University College London, England; 5 Institute of Psychiatry, Psychology and Neuroscience, Kings College London, London, England

## Abstract

**Importance:**

Dysfunctional reward processing is a leading candidate mechanism for the development of certain depressive symptoms, such as anhedonia. However, to our knowledge, there has not yet been a systematic assessment of whether and to what extent depression is associated with impairments on behavioral reward-processing tasks.

**Objective:**

To determine whether depression is associated with impairments in reward-processing behavior.

**Data Sources:**

The MEDLINE/PubMed, Embase, and PsycInfo databases were searched for studies that investigated reward processing using performance on behavioral tasks by individuals with depression and nondepressed control groups, published between January 1, 1946, and August 16, 2019.

**Study Selection:**

Studies that contained data regarding performance by depressed and healthy control groups on reward-processing tasks were included in the systematic review and meta-analysis.

**Data Extraction and Synthesis:**

Summary statistics comparing performance between depressed and healthy groups on reward-processing tasks were converted to standardized mean difference (SMD) scores, from which summary effect sizes for overall impairment in reward processing and 4 subcomponent categories were calculated. Study quality, heterogeneity, replicability-index, and publication bias were also assessed.

**Main Outcome and Measures:**

Performance on reward-processing tasks.

**Results:**

The final data set comprised 48 case-control studies (1387 healthy control individuals and 1767 individuals with major depressive disorder). The mean age was 37.85 years and 58% of the participants were women. These studies used tasks assessing option valuation (n = 9), reward bias (n = 6), reward response vigor (n = 12), reinforcement learning (n = 20), and grip force (n = 1). Across all tasks, depression was associated with small to medium impairments in reward-processing behavior (SMD = 0.345; 95% CI, 0.209-0.480). When examining reward-processing subcomponent categories, impairment was associated with tasks assessing option valuation (SMD = 0.309; 95% CI, 0.147-0.471), reward bias (SMD = 0.644; 95% CI, 0.270-1.017), and reinforcement learning (SMD = 0.352; 95% CI, 0.115-0.588) but not reward response vigor (SMD = 0.083; 95% CI, −0.144 to 0.309). The medication status of the major depressive disorder sample did not explain any of the variance in the overall effect size. There was significant between-study heterogeneity overall and in all subcomponent categories other than option valuation. Significant publication bias was identified overall and in the reinforcement learning category.

**Conclusions and Relevance:**

Relative to healthy control individuals, individuals with depression exhibit reward-processing impairments, particularly for tests of reward bias, option valuation, and reinforcement learning. Understanding the neural mechanisms driving these associations may assist in designing novel interventions.

## Key Points

**Question:**

Are patients with depression associated with impairment on behavioral tests of reward processing compared with healthy control individuals?

**Findings:**

In this systematic review and meta-analysis of data from 48 case-control studies of reward-processing tasks, patients with depression showed a small to medium impairment in reward processing across all tasks. They showed medium to large impairments in reward bias, small to medium impairments in option valuation and reinforcement learning, and small (nonsignificant) impairments in reward response vigor.

**Meaning:**

In this systematic review and meta-analysis, depression is associated with behavioral reward-processing impairments, although this could vary depending on the precise subcomponent measured.

## Teaser

This systematic review and meta-analysis evaluates whether depression is associated with impairments in reward-processing behavior.

## Introduction

Depression is the leading cause of disability worldwide,^1^ and the effectiveness of therapeutic agents for depression is limited.^2^ A lack of detailed understanding of the mechanisms underlying depressive symptoms, such as low mood, fatigue, and anhedonia, is a major barrier to the development of more effective treatment strategies.

It is now well established that depression is associated with disrupted cognitive processing,^3^ for both nonaffective (cold) and affective (hot) information. This includes reward processing,^4,5^ which describes how organisms use reinforcement-related perceptions to guide goal-directed behaviors. A reward-processing framework is especially useful for understanding symptoms associated with motivation, such as reduced interest and activity,^6^ which warrant better understanding because they are associated with poorer outcomes^7,8^ and treatment response.^9^

Reward processing can be divided into a number of subcomponents. According to 1 conceptualization,^6^ reward processing proceeds according to the following sequence of cognitive operations: (1) option generation, the generation of potentially rewarding behavioral options; (2) decision-making, where options are subjected to a cost-benefit evaluation, which balances the utility of potential rewards against associated costs (eg, the potential effort of obtaining those rewards), resulting in the selection of one of the options; (3) anticipation, an anticipatory or preparatory phase associated with physiological arousal before the reward is obtained; (4) action and effort, engagement in action to obtain the reward goal; (5) consummation, the hedonic effect arising from interacting with the reward goal (or alternatively, the frustration of an omitted outcome); and (6) reinforcement learning, learning how to modify behavior in future interactions with similar stimuli using an update signal.

Not all of these subcomponents are straightforward to assess using objective behavioral tasks; anticipation and hedonic impact are typically assessed using physiological responses and self-report, respectively. Over the past 2 decades, reward-processing dysfunction in depression has been the focus of numerous studies, typically using tasks falling into the following 4 categories.^10^

### Option Valuation

Part of subcomponent 2 in the previous section, option valuation describes the process by which individuals evaluate reward-related options when given explicit information about possible options (eg, reward, cost, and probability). An individual’s choice is assumed to reflect the weights that they place on potential rewards and costs (costs may include a potential loss of points/money or the effort needed to obtain the reward).^6^ Studies investigating this domain of reward processing, eg, using the Cambridge Gambling Task, have reported that individuals with depression were less willing than control individuals to place high bets when reward probabilities were high.^11^

### Reward Bias

Also thought to reflect subcomponent 2, reward bias is measured while individuals make difficult decisions (often perceptual) that are rewarded asymmetrically, distinguishing this process from option valuation. Information relating to potential rewards/losses/probabilities is typically not provided explicitly. The reward bias measure, derived from signal detection theory, reflects an individual’s tendency to choose more frequently rewarded stimuli, regardless of perceptual accuracy.^12^ Individuals with depression have been reported to exhibit weaker reward biases than control individuals.^12^

### Reward Response Vigor

Part of subcomponent (4), reward response vigor reflects the speed with which an individual executes an action to obtain a reward. The difference between this and the former 2 types of measure is that here, the measure relates to the actual action taken, not simply the choice to take it. This category includes tasks such as the Monetary Incentive Delay Task^13^ and the Cued Reinforcement Reaction Time Task.^14^

### Reinforcement Learning

Part of subcomponent 6, reinforcement learning describes the process by which an individual uses feedback to change their behavior in the future. Changes in behavior over time are assumed to reflect the updating of value expectations assigned to available behaviors.^6^ Studies using probabilistic learning tasks report that individuals with depression use feedback less effectively than control individuals to accumulate reward.^15^

Three meta-analyses have examined abnormal reward-related neural processing in depression,^16,17,18^ all of which identified lower striatal responses. The striatum, part of the basal ganglia, connects reciprocally with prefrontal areas, (ventral parts of which code stimulus value^19^ and were found to be blunted in 2 of the meta-analyses) as well as the midbrain, which signals the discrepancy between expected and received reward.^20^ Together these areas form part of the brain’s reward circuit, which modulates reward-related behavior and learning.^20^ However, to our knowledge, there has been no meta-analysis of the behavioral reward-processing literature, although several narrative reviews exist.^5,6,10,21,22,23,24,25,26,27^ While narrative reviews can provide a useful overview of the field and an opportunity to develop theoretical accounts, they cannot directly address whether disruptions in reward processing are consistent across samples or quantify the magnitude of identified effects.

Therefore, we aimed to produce a quantitative summary of this literature by conducting a systematic review and meta-analysis of reward-processing behavior in depression. This is an important step in determining whether reward-processing dysfunction is useful for understanding depression. The aims of our meta-analysis were to clarify (1) the nature and extent of differences between depressed and healthy groups on behavioral measures of reward processing and (2) the relative strength and consistency of differences within different reward-processing subdomains.

## Method

### Systematic Review

The Ovid MEDLINE/PubMed, Embase, and PsycInfo databases were searched for articles published between January 1, 1946, and August 16, 2019, inclusive, with titles or abstracts containing the terms (deci* or reward* or motivat* or incentiv* or effort*) and (depress*) and (task* or paradigm* or battery*). The inclusion criteria were as follows: (1) case-control design; (2) included a healthy control group; (3) included a group with major depressive disorder (MDD), assessed according to *DSM-IV/DSM-5* or *International Statistical Classification of Diseases and Related Health Problems, Tenth Revision* criteria; (4) participants were 18 years or older (because there are important differences between developing and adult reward systems)^28^; (5) participants performed a reward-processing task; and (6) task rewards were explicit, ie, money, points, water, or food (we did not include studies that used outcomes that could be considered purely informational, eg, happy/sad faces or variants of correct/incorrect, to ensure specificity). Although it could be argued that these are social rewards, the distinction between purely informational feedback and socially rewarding feedback is unclear in such contexts. A focused search for social reward tasks in depression yielded no eligible studies. The final 2 criteria were (7) samples did not overlap with other included data sets and (8) studies reported data on a behavioral measure of reward processing that could be converted to a case-control standardized mean difference (SMD) score. If articles were otherwise suitable but did not contain such data, the data were requested from the authors.

Where possible, selected behavioral measures related only to reward (ie, not also punishment). For some tasks, this was not possible (because all nonreward outcomes were punishments). Articles were independently assessed by D.C.H. and A.N. Conflicts were highlighted using the Covidence software package (Covidence) and resolved through in-person discussion.

D.C.H. and K.K. rated the included studies on factors that may bias results, using a rating tool based on the Newcastle-Ottawa scale for assessing the quality of nonrandomized studies in meta-analyses (eMethods 1 in the Supplement).^29^ Studies were rated on whether cases and controls were sampled from the same population, how precisely they were defined (eTable 1 in the Supplement), whether they were matched on age, sex, IQ, and personal/household-income or occupation, and whether cases were restricted to only a specific subpopulation (eg, individuals who attempted suicide) (eTable 2 in the Supplement). Where reported, measures of anhedonia or cold cognition were used as continuous moderators, as were the mean age and proportion of women in each study sample. Studies were coded as containing either exclusively unmedicated or at least some medicated cases (eMethods 2 and eTable 3 in the Supplement).

### Meta-analysis

Relevant behavioral measures (eTable 4 in the Supplement) from each study were categorized as measuring option valuation, reward bias, reward response vigor, or reinforcement learning (eTable 5 in the Supplement), then converted to an SMD score and standard error (eMethods 3 and eTable 6 in the Supplement). Within option valuation, reward bias, and reward response vigor, a positive SMD represents a greater response to reward by the control than depressed group (eg, higher weighting of reward in a gambling task, greater reward bias in a signal detection task, or faster response in the rewarded [vs unrewarded] condition of a reaction time task, respectively). Within reinforcement learning, a positive SMD represents faster use of feedback to maximize reward accumulation by the control group than the depressed group (eg, a greater proportion of high-probability reward choices in a 2-arm bandit task). One study assessed the effect of reward on grip force production^30^ and so is excluded from these categories, although included in calculations relating to the overall sample.

The meta-analysis was performed in the statistical packages metafor^31^ and metaviz^32^ (R Statistical Programming) using a restricted maximum likelihood estimator model.^33^ Heterogeneity was assessed using a point estimate of the among-study variance of true effect sizes (*τ^2^*) and the approximate proportion of total variability (*I^2^*).^34^ Sensitivity analysis involved stepwise removal of studies to assess the effect of their removal on effect size and heterogeneity.

Funnel plot asymmetry was assessed using visual inspection of a contour enhanced funnel plot^35^ and the Egger test^36^ (using a 2-sided significance threshold of *P* = .10 because the Egger test has low power when the number of studies is low). Studies missing owing to publication bias were estimated and imputed using the trim-and-fill method. Moderator analyses used random-effects categorical or metaregression models, overall, and in subcomponent categories. Replicability indexes were calculated by reducing the observed median power of studies by the discrepancy between it and the percentage of significant results (within a given category).^37^

## Results

**Figure 1.**
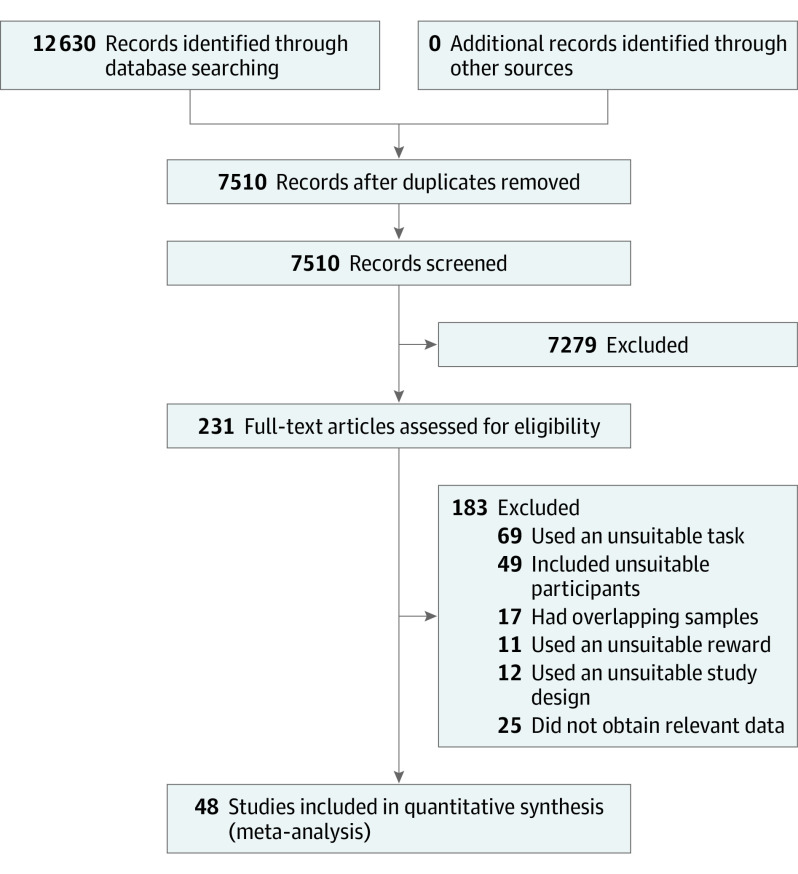
Flow Diagram of Study Selection and Inclusion

Data from 48 studies^11,12,15,30,38,39,40,41,42,43,44,45,46,47,48,49,50,51,52,53,54,55,56,57,58,59,60,61,62,63,64,65,66,67,68,69,70,71,72,73,74,75,76,77,78,79,80^ containing 3154 participants (1387 healthy control individuals and 1767 individuals with MDD) were analyzed. Although we searched primarily for case-control studies, we also attempted to identify relevant longitudinal, population-based studies; none were identified. Figure 1 outlines the search results and the Table outlines the numbers of studies and participants in each category.

**Table.** Search Results for Reward-Processing Categories

Variable	Option valuation	Reward bias	Reward response vigor	Reinforcement learning
No. of studies	9	6	12	20
No. of participants	639	677	499	1291
HC	274	230	249	608
MDD	365	447	250	683

Abbreviations: HC, healthy control individuals; MDD, major depressive disorder.

### Meta-analysis Results

**Figure 2.**
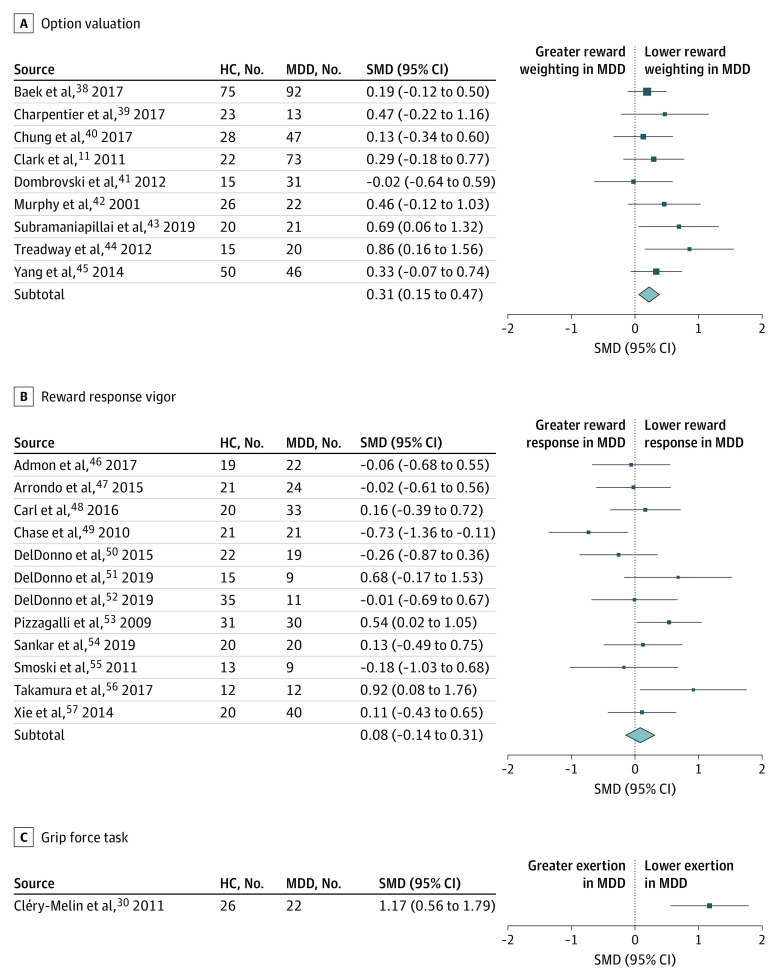
Forest Plot of Option Valuation, Reward Response Vigor, and Grip Force Rectangles and horizontal lines represent, respectively, standardized mean difference (SMD) scores and 95% confidence intervals of individual studies (A, Option valuation studies. B, Reward response vigor studies. C, Grip force task). Diamonds represent the summary effects and 95% confidence intervals for the respective reward processing subcomponent category. HC indicates healthy control; MDD, major depressive disorder.

**Figure 3.**
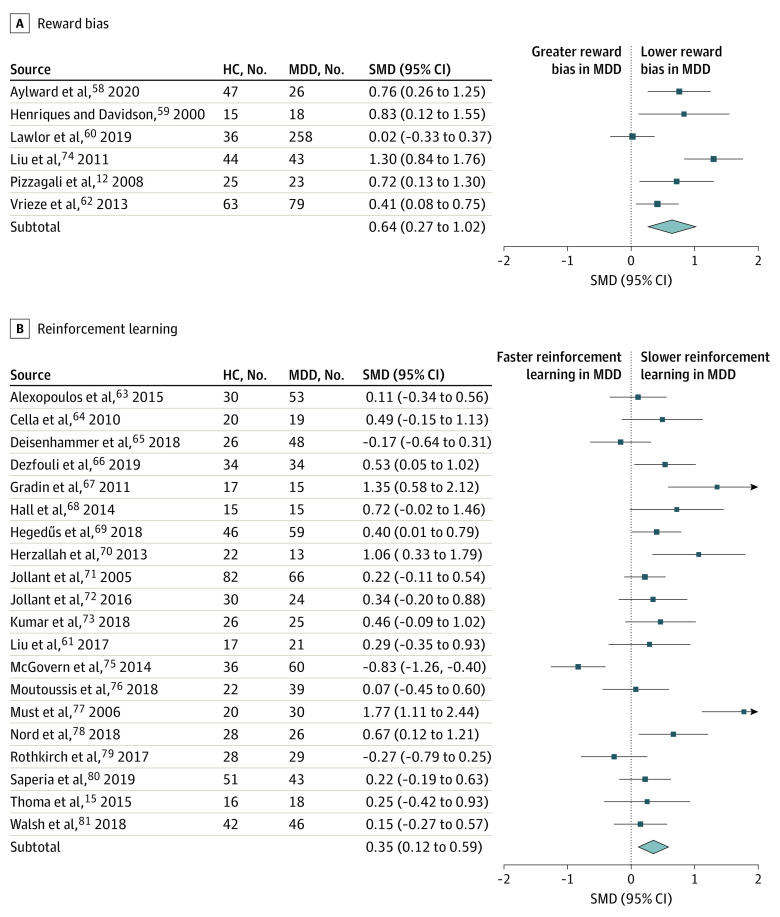
Forest Plot of Reward Bias and Reinforcement Learning Rectangles and horizontal lines represent, respectively, standardized mean difference (SMD) scores and 95% confidence intervals of individual studies (A, Reward bias studies. B, Reinforcement learning studies). Diamonds represent the summary effects and 95% confidence intervals for the respective reward processing subcomponent category. HC indicates healthy control; MDD, major depressive disorder.

Across all studies, there was a small to medium reward processing impairment in depressed compared to healthy groups (SMD, 0.345; 95% CI, 0.209-0.480). Analysis of the 4 subcomponent categories (Table; Figure 2 and Figure 3) revealed a small to medium impairment in option valuation (SMD, 0.309; 95% CI, 0.147-0.471), a medium to large impairment in reward bias (SMD, 0.644; 95% CI, 0.270-1.017), a small to negligible (nonsignificant) impairment in reward response vigor (SMD, 0.083; 95% CI, −0.144 to 0.309) and a small to medium impairment in reinforcement learnin*g* (SMD, 0.352; 95% CI, 0.115-0.588).

Overall interstudy heterogeneity was substantial (*τ^2^* = 0.15; *I^2^* = 68%). There was no interstudy heterogeneity in the option valuation category (*τ^2^* = 0.0; *I^2^* = 0%). There was low to moderate heterogeneity in the reward response vigor category (*τ^2^* = 0.05; *I^2^* = 34%). There was substantial to considerable heterogeneity in the reward bias (*τ^2^* = 0.16; *I^2^* = 75%) and reinforcement learning (*τ^2^* = 0.21; *I^2^* = 76%) categories. Excluding 1 study^59^ reduced heterogeneity in the reward bias category to moderate to substantial (*τ^2^* = 0.08; *I^2^* = 55%) and increased the effect size to large (SMD, 0.784; 95% CI, 0.453-1.116). Excluding 1 study^14^ eliminated heterogeneity in the reward response vigor category (τ^2^ = 0.0; I^2^ = 0%); the effect size remained small to negligible and nonsignificant (SMD, 0.157; 95% CI, −0.033 to 0.347). Removing individual studies did not reduce interstudy heterogeneity either in the other categories or in the overall sample.

### Moderator Analysis

Potential sources of bias, including the reward processing categories, were assessed as moderators. Of the total variation in effect sizes, 68% was owing to between-study differences. The medication status of the MDD sample (unmedicated vs at least partially medicated) explained 0% of the variance in the global effect size, with no effect of medication status in any sub-component category.

In those studies that reported summary statistics of anhedonia scores (n = 18) or cold-cognitive task performance (n = 8), SMDs of respective measures used as continuous moderators revealed no significant effects of either on reward processing (too few studies were available to perform meaningful analyses in subcomponent categories).

Studies including an exclusively elderly sample (n = 4) yielded a smaller (*P* < .001) and nonsignificant effect (SMD, −0.127; 95% CI, −0.555 to 0.300) than those including nonelderly samples (n = 44; SMD, 0.390; 95% CI, 0.254-0.525). Studies that clearly matched groups for sex yielded a larger effect than those that did not (eResults in the Supplement); however, there was no moderation effect of the proportion of female participants in study samples. All other moderator analyses of the overall sample yielded nonsignificant results (eResults in the Supplement).

### Publication Bias

**Figure 4.**
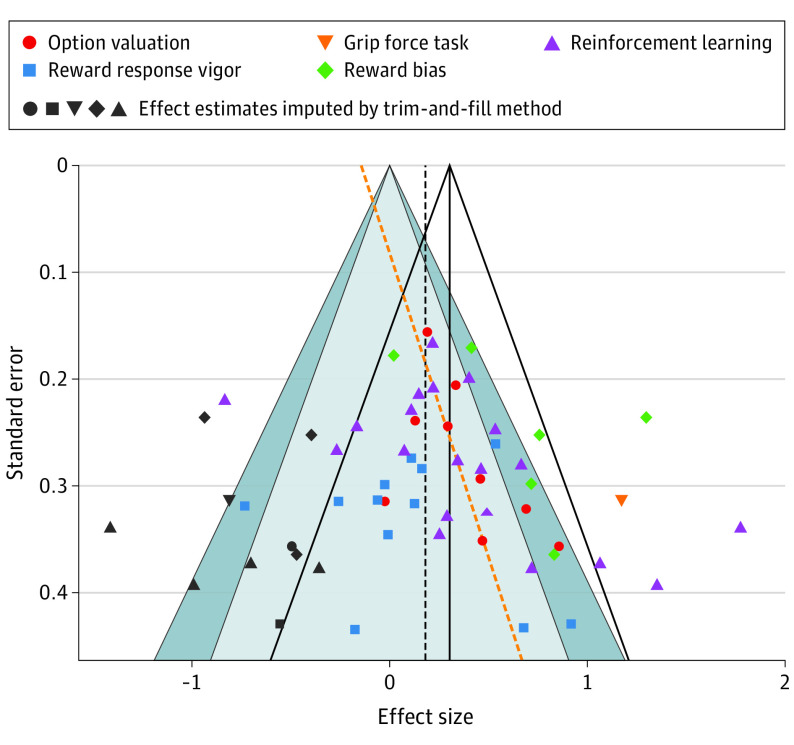
Contour-Enhanced Funnel Plot The solid black vertical line represents the observed overall summary effect. The unfilled funnel represents 95% confidence intervals for individual effect estimates, assuming no bias. The dashed black vertical line represents the summary effect when including imputed studies (using the trim-and-fill method). The dashed orange line represents the Egger regression line. The light blue funnel represents the 95% confidence intervals and the dark blue funnel represents the 99% confidence intervals for individual effect estimates, including imputed studies. The colored shapes represent individual included effect estimates: red circles for option valuation, blue squares for reward response vigor, orange inverted triangle for the single grip force task, green diamonds for reward bias, and purple triangles for reinforcement learning. All dark gray shapes represent effect estimates imputed by trim-and-fill.

Overall publication bias was significant (Egger test: *z* = 2.082; *P* = .04; Figure 4); however, genuine between-study heterogeneity may be mistaken for publication bias.^81^ Overall median power and *R* index were low (median power, 22%; *R* index, 7%). It was not possible to assess publication bias, median power, or *R* index scores in the option valuation or reward bias categories owing to the low number of studies in both. Publication bias was significant in the reinforcement learning category (*z* = 3.092; *P* = .002) and nonsignificant in the reward response vigor category. Median power and *R* index scores were low in both reward response vigor (median power, 6%; *R* index, 0%) and reinforcement learning (median power, 25%; *R* index, 16%) categories.

## Discussion

To our knowledge, this is the first systematic review and meta-analysis to quantify behavioral reward-processing differences between depressed and healthy groups. Across 48 studies, we found that depressed groups had small to medium (SMD, 0.345) reward-processing impairments relative to healthy groups. However, there were potentially important differences between reward-processing subcomponents.

The largest impairment was observed in the reward bias category (SMD, 0.644), which is unlikely to be owing to impairment in non–reward-based processing (eg, perceptual) because the signal detection theory discrimination measure did not differ significantly between the groups in most included studies. From this meta-analysis, the reward bias impairment in depression appears most robust. Prior computational analysis suggests that this may be driven by lower reward valuation.^82^

The reinforcement learning category yielded a small to medium effect (SMD, 0.352). Many tasks in this category could not allow reward response to be disentangled from confounds such as working memory impairment.^83^ Future studies that investigate reinforcement learning impairments in depression may benefit from using tasks that allow the contributions of hot and cold cognitive impairment to be dissociated.^83^

Individuals with depression exhibited a small to medium impairment in option valuation (SMD, 0.309), which is associated with “decisional anhedonia” resulting from impaired cost-benefit decision-making.^24^ The reward response vigor category exclusively contained tasks measuring reaction times, which may be vulnerable to attentional confounds. While these cannot be fully controlled for, we go some way toward doing so by including only tasks that compared responding in more vs less rewarded conditions. While reward response vigor yielded a nonsignificant summary effect (SMD, 0.083), the single study^30^ that assessed grip force production yielded a large effect (SMD, 1.17). Speculatively, this might indicate that reward-processing impairments in depression are associated more with fatigability than the speed of action, consistent with the higher prevalence of anergia than psychomotor retardation in depression.^84^ Further research on the effect of reward on grip force in depression is warranted.

*R* index values suggest that significant results may be difficult to replicate. However, the *R* index is conservative and less precise when true power is low.^37^ Assuming that future studies can expect to yield effect sizes comparable with the overall summary effect in this meta-analysis (SMD, 0.345), they will require sample sizes of 133 per group to achieve a power of 0.8 at a significance of .05 (2-tailed). This is considerably larger than the mean sample size in the included studies (33 per group).

Moderator analysis revealed no significant association for between-study variation and either anhedonia or cold cognitive performance on reward-processing impairment. These results should be interpreted cautiously owing to the low number of studies that could be included and heterogeneity in measuring these constructs. The assessment of within-study variation in such factors would have been more informative, but the necessary correlation coefficients were rarely reported; future studies should report correlations between reward processing and anhedonia and/or cold cognitive impairment. Those studies that recruited exclusively elderly participants yielded a nonsignificant summary effect (SMD, −0.127). Speculatively, this may be owing to the effect of healthy aging on reward processing.^85^ None of the included studies controlled for personal/household income, a potentially important oversight in studies that use monetary rewards because depression is associated with lower household income^86^ (and, by extension, a higher utility of money)^87^ than the general population. Future studies that use monetary rewards should assess income levels.

### Limitations

Several limitations of our analyses merit comment. First, reward processing comprises a heterogeneous set of processes, which we categorized according to 4 subcomponents. However, there are several potential ways to measure function in each category. Therefore, this meta-analysis sometimes combines dissimilar measures in its summary statistics. For example, option valuation contains studies that probe the effect of reward on the willingness to exert effort (in 3 cases) and to take risks (in 6 cases). Second, the medicated samples were often not entirely medicated, used a variety of medications (even within-study), and at different doses. Therefore, the nonsignificant moderation result for medication status is difficult to interpret. The effect of medication on reward processing in depression is best investigated in the context of randomized clinical trials. Third, we did not investigate response to punishment (because very few of the studies investigated responses to punishment separately from reward). Given the importance of sensitivity to punishment in some cognitive models of depression,^88^ this is an important omission. Fourth, there was significant heterogeneity, overall and in all categories except option valuation, making the interpretation of the summary effects less clear. Fifth, there was significant publication bias overall and in reinforcement learning particularly, resulting in a potential overestimation of the summary effects. Sixth, we were unable to examine 2 important reward-processing components, the anticipation and hedonic effect of rewards. A literature search for studies in these categories yielded 5 studies,^89,90,91,92,93^ which investigated the hedonic effect of oral sucrose solutions in healthy vs depressed groups. However, of these studies, too few contained suitable data for the meta-analysis to include a hedonic effect category. Seventh, this systematic review and meta-analysis summarizes the findings of case-control studies, which do not inform us about the causal relationship between reward-processing impairment and depression or its treatment. Longitudinal studies examining reward processing in depression are needed to answer these important questions. Eighth, the effect size estimates were unadjusted for covariates and so may be affected by confounds.

## Conclusions

Conducting a meta-analysis of 48 studies, we found that depression was reliably associated with small to medium reward-processing impairments overall and of varying magnitudes across several reward-processing subdomains. This is important because the cognitive and neural mechanisms underlying reward processing and its subdomains are relatively well understood.^24^ Research on reward processing may therefore be a credible route to better characterizing mechanistic heterogeneity within depression, as well as potentially highlighting novel targets for treatment.

## Notes

Supplement. eMethods 1. Modified version of the Newcastle-Ottawa scale for assessing the quality of nonrandomized studies in meta-analyses, used to assess potential sources of bias. **eTable 1.** Quality ratings for ‘selection’ component of the above rating scale **eTable 2.** Quality ratings for ‘comparability’ component above **eMethods 2.** List of additional moderators used to assess the potential sources of bias **eTable 3.** Additional moderators used to assess the potential sources of bias **eTable 4.** Tasks and measures included in the meta-analysis **eTable 5.** Reward processing categories into which the studies were categorised, medication status of the depressed groups and sample sizes **eMethods 3.** Formulae used to convert study measures into Cohen’s ds and associated variances **eTable 6.** The summary statistic(s) that were converted to a Cohen’s d and the variance on d for each included study **eResults.** Moderator analyses
