# Supplementary material for: Reward-Processing Behavior in Depressed Participants Relative to Healthy Volunteers: A Systematic Review and Meta-analysis
Source: JAMA Psychiatry. 2020 Jul 29;77(12):1286–95. doi: 10.1001/jamapsychiatry.2020.2139 (PMC7391183; doi:10.1001/jamapsychiatry.2020.2139)
Supplement: Supplement. — eMethods 1. Modified version of the Newcastle-Ottawa scale for assessing the quality of nonrandomized studies in meta-analyses, used to assess potential sources of bias. eTable 1. Quality ratings for ‘selection’ component of the above rating scale eTable 2. Quality ratings for ‘comparability’ component above eMethods 2. List of additional moderators used to assess the potential sources of bias eTable 3. Additional moderators used to assess the potential sources of bias eTable 4. Tasks and measures included in the meta-analysis eTable 5. Reward processing categories into which the studies were categorised, medication status of the depressed groups and sample sizes eMethods 3. Formulae used to convert study measures into Cohen’s ds and associated variances eTable 6. The summary statistic(s) that were converted to a Cohen’s d and the variance on d for each included study eResults. Moderator analyses [file jamapsychiatry-e202139-s001.pdf]

Supplementary Online Content

Halahakoon DC, Kieslich K, O'Driscoll C, Nair A, Lewis G, Roiser JP. Association of depression with reward-processing behavior: a systematic review and meta-analysis. *JAMA Psych*. Published online July 29, 2020. doi:10.1001/jamapsychiatry.2020.2139

eMethods 1. Modified version of the Newcastle-Ottawa scale for assessing the quality of nonrandomized studies in meta-analyses, used to assess potential sources of bias.
eTable 1. Quality ratings for 'selection' component of the above rating scale
eTable 2. Quality ratings for 'comparability' component above
eMethods 2. List of additional moderators used to assess the potential sources of bias
eTable 3. Additional moderators used to assess the potential sources of bias
eTable 4. Tasks and measures included in the meta-analysis
eTable 5. Reward processing categories into which the studies were categorised, medication status of the depressed groups and sample sizes
eMethods 3. Formulae used to convert study measures into Cohen's ds and associated variances
eTable 6. The summary statistic(s) that were converted to a Cohen's d and the variance on d for each included study
eResults. Moderator analyses

This supplementary material has been provided by the authors to give readers additional information about their work.

© 2020 American Medical Association. All rights reserved.

eMethods 1. Modified version of the Newcastle-Ottawa Scale for Assessing the Quality of Nonrandomized Studies in Meta-Analyses, used to assess potential sources of bias.

Potential sources of bias were assessed using a modified version of the Newcastle-Ottawa Scale for Assessing the Quality of Nonrandomized Studies in Meta-Analyses. The studies are scored on:

Selection

1. **MDD Definition**: Is the case definition adequate?
   - A) Cases were defined as MDD according DSM or ICD criteria according to a validated assessment tool or by an experienced clinician
   - B) Cases were defined as MDD according DSM or ICD criteria but the method for assessing MDD status was not stated.
   - C) Cases were described as ‘clinically depressed’ but no further description was given.
2. **MDD Generality**: Was a General sample of cases tested?
   - A) A General sample of MDD was tested.
   - B) Recruitment of MDD cases was restricted to a specific sub-sample.
3. **HC Selection**: Selection of Controls
   - A) Controls were selected from the same population as cases
   - B) Controls were not selected from the same population as cases
   - C) No description
4. **HC Definition**: Definition of Controls
   - A) HC were defined clearly defined as having no current or past psychopathology
   - B) Controls were not clearly defined as having no current or past psychopathology.

Comparability (Comparability of cases and controls on the basis of the design or analysis)

1. **Does the study control for Age**: Yes/No/Unclear
2. **Does the study control for Gender**: Yes/No/Unclear
3. **Does the study control for IQ**: Yes/No/Unclear
4. **Does the study control for Personal or Household Income/Occupation**: Yes/No/Unclear

© 2020 American Medical Association. All rights reserved.

eTable 1. Quality ratings for 'Selection' component of the above rating scale.

Author, Year	Selection			
	MDD Definition	MDD Generality	HC selection	HC definition
Baek, 2017[1]	A	B (suicide attempters)	B	A
Charpentier, 2017[2]	A	A	A	B
Chung, 2017[3]	A	A	C	B
Clarke, 2011[4]	A	B (elderly, suicide attempters)	B	A
Dombrovski, 2012[5]	A	B (elderly, suicide attempters)	C	A
Murphy, 2001[6]	A	A	B	B
Subramaniapillai, 2019[7]	A	A	C	A
Treadway, 2012[8]	A	A	B	A
Yang, 2014[9]	A	A	B	B
Admon, 2017[10]	A	A	A	A
Arrondo, 2015[11]	A	A	B	B
Carl, 2016[12]	A	A	B	B
Chase, 2010[13]	A	A	B	A
DelDonno, 2015[14]	A	A	A	A
DelDonno, 2019a[15]	A	A	A	A
DelDonno, 2019b[16]	A	A	A	A
Pizzagalli, 2009[17]	A	A	B	A
Sankar, 2019[18]	A	B (women only)	A	A
Smoski, 2011[19]	A	A	B	B
Takamura, 2017[20]	A	A	B	A
Xie, 2014[21]	A	A	B	A
Clery-Melin, 2011[22]	A	A	B	A
Aylward, 2019[23]	A	A	A	A
Henriques, 2000[24]	A	A	A	A
Lawlor, 2019[25]	A	A	B	B
Liu, 2011[26]	A	A	B	B
Pizzagali, 2008[27]	A	A	B	A
Vrieze, 2013[28]	A	A	B	A
Alexopoulos, 2015[29]	A	B (elderly)	A	A
Cella, 2010[30]	A	A	B	A
Deisenhammer, 2018[31]	A	B (suicide attempters)	B	B
Dezfouli, 2019[32]	A	A	B	A
Gradin, 2011[33]	A	A	A	B
Hall, 2014[34]	A	A	B	A
Hegedus, 2018[35]	A	B (suicide attempters)	B	A
Herzallah, 2013[36]	A	B (melancholic)	B	A
Jollant, 2005[37]	A	B (suicide attempters)	C	A
Jollant, 2016[38]	A	A	C	A
Kumar, 2018[39]	A	A	A	A
Liu, 2017[40]	A	A	B	A
McGovern, 2014[41]	A	B (elderly)	A	A
Moutoussis, 2018[42]	A	A	B	B
Must, 2006[43]	A	A	B	A
Nord, 2018[44]	A	A	B	A
Rothkirch, 2017[45]	A	A	B	A
Saperia, 2019[46]	A	A	C	A
Thoma, 2015[47]	A	A	B	A
Walsh, 2018[48]	A	A	A	A

MDD=Depressed Group; HC=Healthy Control Group.
Please refer to the above rating tool for the meanings of 'MDD definition', 'MDD Generality', 'HC selection' and 'HC definition' as well as 'A','B' and 'C' in each column. In question 2 (MDD Generality), where recruitment was restricted only to a specific subsample of MDD patients, this subsample is specified in parentheses.

© 2020 American Medical Association. All rights reserved.

eTable 2. Quality ratings for 'Comparability' component above.

Author, Year	Comparability			
	Age	Gender	IQ	Inc/Occ
Baek, 2017[1]	Y	Y	N	N
Charpentier, 2017[2]	Y	Y	Y	N
Chung, 2017[3]	Y	N	Y	N
Clarke, 2011[4]	Y	Y	N	N
Dombrovski, 2012[5]	Y	Y	Y	N
Murphy, 2001[6]	Y	Y	Y	N
Subramaniapillai, 2019[7]	Y	Y	N	N
Treadway, 2012[8]	Y	Y	N	N
Yang, 2014[9]	Y	Y	N	N
Admon, 2017[10]	Y	Y	N	N
Arrondo, 2015[11]	Y	Y	Y	N
Carl, 2016[12]	Y	Y	Y	N
Chase, 2010[13]	Y	Y	Y	N
DelDonno, 2015[14]	Y	Y	Y	N
DelDonno, 2019a[15]	N	Y	Y	N
DelDonno, 2019b[16]	Y	Y	Y	N
Pizzagalli, 2009[17]	Y	Y	N	N
Sankar, 2019[18]	Y	Y	N	N
Smoski, 2011[19]	Y	Y	Y	N
Takamura, 2017[20]	Y	Y	Y	N
Xie, 2014[21]	Y	Y	N	N
Clery-Melin, 2011[22]	Y	Y	N	N
Aylward, 2019[23]	Y	Y	Y	N
Henriques, 2000[24]	Y	Y	N	N
Lawlor, 2019[25]	Y	Y	N	N
Liu, 2011[26]	Y	Y	Y	N
Pizzagali, 2008[27]	Y	Y	N	N
Vrieze, 2013[28]	Y	Y	N	N
Alexopoulos, 2015[29]	Y	N	N	N
Cella, 2010[30]	Y	Y	N	N
Deisenhammer, 2018[31]	Y	Y	N	N
Dezfouli, 2019[32]	Y	Y	Y	N
Gradin, 2011[33]	Y	Y	Y	N
Hall, 2014[34]	Y	Y	N	N
Hegedus, 2018[35]	Y	Y	N	N
Herzallah, 2013[36]	Y	Y	N	N
Jollant, 2005[37]	Y	N	Y	N
Jollant, 2016[38]	Y	Y	Y	N
Kumar, 2018[39]	Y	Y	N	N
Liu, 2017[40]	Y	Y	Y	N
McGovern, 2014[41]	Y	U	N	N
Moutoussis, 2018[42]	Y	Y	N	N
Must, 2006[43]	Y	Y	N	N
Nord, 2018[44]	Y	Y	Y	N
Rothkirch, 2017[45]	Y	Y	N	N
Saperia, 2019[46]	Y	Y	N	N
Thoma, 2015[47]	Y	Y	Y	N
Walsh, 2018[48]	Y	Y	Y	N

IQ=Intelligence Quotient; Inc/Occ= Personal or Household Income/Occupation; Y=case and control groups were controlled for on the relevant measure; N= case and control groups were not controlled for on the relevant measure; U=unclear whether case and control groups were controlled for on the relevant measure.

© 2020 American Medical Association. All rights reserved.

eMethods 2. List of additional moderators used to assess the potential sources of bias.

In addition to those listed in eTables 1 and 2, other potential sources of bias were assessed using the following moderators:

- **Reward Processing Category**: Which of the four reward processing categories the studies belonged to.
- **Medication Status of MDD Group**: Whether the entire study sample was unmedicated.
- **Proportion of Females**: The proportion of female participants in the total study sample.
- **Mean Age**: The mean age of participants in the total study sample.
- **Risk-based OV task**: Within the Option Valuation category, whether the reward processing task was risk-based.
- **Difference in Anhedonia Score**: The difference in anhedonia questionnaire score between cases and controls (using standardized mean differences).
- **Difference in Cold Cognitive Task Performance**: The difference in performance on a cold cognitive task between cases and controls (using standardized mean differences).

© 2020 American Medical Association. All rights reserved.

eTable 3. Additional moderators used to assess the potential sources of bias.

Author, Year	Reward Processing Category	Medication Status of MDD Group	Proportion of Females	Mean Age	Risk-based OV task	Difference in Anhedonia Score		Difference in Cold Cognitive Task Performance	
						d	var_d	d	var_d
Baek, 2017[1]	OV	M	0.449	24.2	Y	NA	NA	NA	NA
Charpentier, 2017[2]	OV	U	0.790	25.47	Y	NA	NA	NA	NA
Chung, 2017[3]	OV	M	0.684	35.46	Y	2.046	0.067	NA	NA
Clarke, 2011[4]	OV	M	0.495	68.5	Y	NA	NA	NA	NA
Dombrovski, 2012[5]	OV	M	0.543	68.675	Y	NA	NA	NA	NA
Murphy, 2001[6]	OV	M	0.563	37.9	Y	NA	NA	NA	NA
Subramaniapillai, 2019[7]	OV	M	0.537	39.985	N	1.177	0.115	0.470	0.100
Treadway, 2012[8]	OV	M	0.657	41.05	N	1.936	0.170	NA	NA
Yang, 2014[9]	OV	M	0.448	26.63	N	1.351	0.051	NA	NA
Admon, 2017[10]	RRV	U	0.723	25.8	NA	1.573	0.128	NA	NA
Arrondo, 2015[11]	RRV	M	0.244	33.705	NA	1.432	0.112	NA	NA
Carl, 2016[12]	RRV	U	0.679	32.15	NA	NA	NA	NA	NA
Chase, 2010[13]	RRV	M	0.452	46.31	NA	NA	NA	NA	NA
DelDonno, 2015[14]	RRV	U	0.760	29.015	NA	1.351	0.120	0.677	0.104
DelDonno, 2019a[15]	RRV	U	0.775	27.12	NA	1.051	0.201	NA	NA
DelDonno, 2019b[16]	RRV	M	0.645	22.92	NA	NA	NA	NA	NA
Pizzagalli, 2009[17]	RRV	U	0.459	40.985	NA	NA	NA	NA	NA
Sankar, 2019[18]	RRV	M	1.000	30.125	NA	NA	NA	NA	NA
Smoski, 2011[19]	RRV	M	NA	30.3	NA	NA	NA	NA	NA
Takamura, 2017[20]	RRV	M	0.500	41.15	NA	NA	NA	NA	NA
Xie, 2014[21]	RRV	M	0.667	31.915	NA	NA	NA	NA	NA
Clery-Melin, 2011[22]	(no category)	M	0.708	44.05	NA	NA	NA	NA	NA
Aylward, 2019[23]	RB	U	0.627	28.88	NA	NA	NA	0.283	0.060
Henriques, 2000[24]	RB	U	0.606	33.055	NA	NA	NA	0.831	0.133
Lawlor, 2019[25]	RB	U	0.656	37.03	NA	3.337	0.051	0.333	0.032
Liu, 2011[26]	RB	U	0.540	27.8	NA	0.889	0.051	NA	NA
Pizzagali, 2008[27]	RB	U	0.438	41.005	NA	3.756	0.230	NA	NA
Vrieze, 2013[28]	RB	M	0.606	44.75	NA	2.506	0.051	NA	NA
Alexopoulos, 2015[29]	RL	U	NA	72.505	NA	NA	NA	0.115	0.052
Cella, 2010[30]	RL	M	0.462	35.45	NA	NA	NA	NA	NA
Deisenhammer, 2018[31]	RL	M	0.670	39.975	NA	NA	NA	NA	NA
Dezfouli, 2019[32]	RL	M	0.559	22.6	NA	NA	NA	NA	NA
Gradin, 2011[33]	RL	M	0.594	42.955	NA	NA	NA	NA	NA

Please refer to the list above for the description of the moderators.
OV=Option Valuation; RV=Response Vigour; RB=Reward Bias; RL=Reinforcement Learning; MDD=Depressed Group; U=Entirely Unmedicated Depressed Group; M=At Least Partially Medicated Depressed Group; Y=the Reward Processing OV Task was risk-based; N=the Reward Processing OV task was not risk-based; d=Cohen's d; Var_d=Variance on d.

© 2020 American Medical Association. All rights reserved.

eTable 3. Additional moderators used to assess the potential sources of bias (continued).

Author, Year	Reward Processing Category	Medication Status of MDD Group	Proportion of Females	Mean Age	Risk-based OV task	Difference in Anhedonia Score		Difference in Cold Cognitive Task Performance	
						d	var_d	d	var_d
Hall, 2014[34]	RL	M	0.700	47	NA	NA	NA	NA	NA
Hegedus, 2018[35]	RL	U	0.638	34.985	NA	NA	NA	NA	NA
Herzallah, 2013[36]	RL	U	NA	27.865	NA	NA	NA	NA	NA
Jollant, 2005[37]	RL	M	0.405	40.14	NA	NA	NA	NA	NA
Jollant, 2016[38]	RL	U	0.593	35.5	NA	NA	NA	-0.301	0.076
Kumar, 2018[39]	RL	U	0.745	25.78	NA	3.395	0.191	NA	NA
Liu, 2017[40]	RL	U	0.540	29.5	NA	1.009	0.120	NA	NA
McGovern, 2014[41]	RL	U	NA	71.625	NA	NA	NA	NA	NA
Moutoussis, 2018[42]	RL	M	0.541	33.66	NA	NA	NA	NA	NA
Must, 2006[43]	RL	M	0.580	43.15	NA	NA	NA	NA	NA
Nord, 2018[44]	RL	U	0.426	27.375	NA	1.771	0.103	NA	NA
Rothkirch, 2017[45]	RL	U	0.638	36.225	NA	1.997	0.105	NA	NA
Saperia, 2019[46]	RL	M	0.521	32	NA	1.330	0.052	-0.317	0.043
Thoma, 2015[47]	RL	M	0.529	41.555	NA	NA	NA	NA	NA
Walsh, 2018[48]	RL	U	0.705	29.865	NA	2.874	0.092	NA	NA

Please refer to the list above for the description of the moderators.
OV=Option Valuation; RV=Response Vigour; RB=Reward Bias; RL=Reinforcement Learning; MDD=Depressed Group; U=Entirely Unmedicated Depressed Group; M=At Least Partially Medicated Depressed Group; Y=the Reward Processing OV Task was risk-based; N=the Reward Processing OV task was not risk-based; d=Cohen's d; Var_d=Variance on d.

© 2020 American Medical Association. All rights reserved.

eTable 4. Tasks and Measures Included in the Meta-Analysis.

Author, Year	Task	Measure
Baek, 2017[1]	Gambling Task	Risk Aversion in Gain Condition
Charpentier, 2017[2]	Gambling Task	Risk Aversion
Chung, 2017[3]	Risky Decision Making task	Risk Preference
Clarke, 2011[4]	Cambridge Gambling Task	Risk Adjustment
Dombrovski, 2012[5]	Cambridge Gambling Task	Risk Adjustment
Murphy, 2001[6]	Cambridge Gambling Task	Risk Adjustment
Subramaniapillai, 2019[7]	Effort Expenditure for Reward Task	Mean Proportion trials in which High Cost/High Reward chosen
Treadway, 2012[8]	Effort Expenditure for Reward Task	Mean Proportion trials in which High Cost/High Reward chosen
Yang, 2014[9]	Effort Expenditure for Reward Task	Mean Proportion trials in which High Cost/High Reward chosen
Admon, 2017[10]	Monetary Incentive Delay	Neutral Reaction Time minus Reward Reaction Time
Arrondo, 2015[11]	Monetary Incentive Delay	Neutral Reaction Time minus Reward Reaction Time
Carl, 2016[12]	Monetary Incentive Delay	Group x Trial interaction; Reaction time
Chase, 2010[13]	Choice Reinforcement Reaction Time Task	Winnings
DelDonno, 2015[14]	Monetary Incentive Delay	Neutral Reaction Time minus Reward Reaction Time
DelDonno, 2019a[15]	Monetary Incentive Delay	Neutral Reaction Time minus Reward Reaction Time
DelDonno, 2019b[16]	Monetary Incentive Delay	Neutral Reaction Time minus Reward Reaction Time
Pizzagalli, 2009[17]	Monetary Incentive Delay	Neutral Reaction Time minus Reward Reaction Time
Sankar, 2019[18]	Monetary Incentive Delay	Neutral Reaction Time minus Reward Reaction Time
Smoski, 2011[19]	Monetary Incentive Delay	Neutral Reaction Time minus Reward Reaction Time
Takamura, 2017[20]	Monetary Incentive Delay	Neutral Reaction Time minus Reward Reaction Time
Xie, 2014[21]	Monetary Incentive Delay	Neutral Reaction Time minus Reward Reaction Time
Clery-Melin, 2011[22]	Grip Force Task	Force Production
Aylward, 2019[23]	Tone Identification Task	Response Bias
Henriques, 2000[24]	Signal Detection Task (words)	Response Bias, Neutral vs Reward Condition
Lawlor, 2019[25]	Probabilistic Reward Task	Response Bias, 2nd block
Liu, 2011[26]	Probabilistic Reward Task	Response Bias, block 3, no-stress condition
Pizzagali, 2008[27]	Probabilistic Reward Task	Response Bias, block 3
Vrieze, 2013[28]	Probabilistic Reward Task	Response Bias, block 3
Alexopoulos, 2015[29]	Iowa Gambling Task	Advantageous Decks minus Disadvantageous Decks
Cella, 2010[30]	Iowa Gambling Task	Advantageous Decks minus Disadvantageous Decks, phase 1, last block
Deisenhammer, 2018[31]	Iowa Gambling Task	Advantageous Decks minus Disadvantageous Decks
Dezfouli, 2019[32]	Instrumental Learning Task	Proportion, Higher Reward Probability Option Chosen
Gradin, 2011[33]	Instrumental Learning Task	Number of Scores

© 2020 American Medical Association. All rights reserved.

eTable 4. Tasks and Measures Included in the Meta-Analysis (continued).

Author, Year	Task	Measure
Hall, 2014[34]	Contingency Reversal Reward Paradigm	Number of Successful Switches
Hegedus, 2018[35]	Iowa Gambling Task	ABCD deck, block 5 score (Advantageous Decks minus Disadvantageous Decks)
Herzallah, 2013[36]	Instrumental Learning Task	% of Correct Responses in Block 4 for Reward Trials
Jollant, 2005[37]	Iowa Gambling Task	Advantageous Decks minus Disadvantageous Decks, last block
Jollant, 2016[38]	Iowa Gambling Task	Advantageous Decks minus Disadvantageous Decks in second half of task (last 50 trials)
Kumar, 2018[39]	probabilistic instrumental learning task	% Choice Accuracy
Liu, 2017[40]	Reinforcement Learning task	% High-Probability Stimuli Chosen (reward trials)
McGovern, 2014[41]	Iowa Gambling Task	Advantageous Decks minus Disadvantageous Decks, last block
Moutoussis, 2018[42]	Go/No-Go task	Appetitive Motivational Exchange Rate for Rewards
Must, 2006[43]	Iowa Gambling Task	Advantageous Decks minus Disadvantageous Decks, first phase, last block
Nord, 2018[44]	Go/No-Go task	Probability of Making a ‘Go’ response
Rothkirch, 2017[45]	Reinforcement Learning Task	Optimal Choice Rate, 5th block, Reward Trials
Saperia, 2019[46]	Iowa Gambling Task	Advantageous Decks minus Disadvantageous Decks, last block.
Thoma, 2015[47]	Probabilistic Selection Task (active)	% Correct Responses, block 4
Walsh, 2018[48]	Probabilistic Instrumental Learning Task	Money Won in Win Trials at Baseline Session

© 2020 American Medical Association. All rights reserved.

eTable 5. Reward Processing Categories into Which the Studies were Categorised, Medication Status of the Depressed Group and Sample Sizes.

Author, Year	Reward Processing Category	Sample Size (HC)	Sample Size (MDD)
Baek, 2017[1]	OV	75	92
Charpentier, 2017[2]	OV	23	13
Chung, 2017[3]	OV	28	47
Clarke, 2011[4]	OV	22	73
Dombrovski, 2012[5]	OV	15	31
Murphy, 2001[6]	OV	26	22
Subramaniapillai, 2019[7]	OV	20	21
Treadway, 2012[8]	OV	15	20
Yang, 2014[9]	OV	50	46
Admon, 2017[10]	RRV	19	22
Arrondo, 2015[11]	RRV	21	24
Carl, 2016[12]	RRV	20	33
Chase, 2010[13]	RRV	21	21
DelDonno, 2015[14]	RRV	22	19
DelDonno, 2019a[15]	RRV	15	9
DelDonno, 2019b[16]	RRV	35	11
Pizzagalli, 2009[17]	RRV	31	30
Sankar, 2019[18]	RRV	20	20
Smoski, 2011[19]	RRV	13	9
Takamura, 2017[20]	RRV	12	12
Xie, 2014[21]	RRV	20	40
Aylward, 2019[23]	RB	47	26
Henriques, 2000[24]	RB	15	18
Lawlor, 2019[25]	RB	36	258
Liu, 2011[26]	RB	44	43
Pizzagali, 2008[27]	RB	25	23
Vrieze, 2013[28]	RB	63	79
Clery-Melin, 2011[22]	(no category)	26	22
Alexopoulos, 2015[29]	RL	30	53
Cella, 2010[30]	RL	20	19
Deisenhammer, 2018[31]	RL	26	48
Dezfouli, 2019[32]	RL	34	34
Gradin, 2011[33]	RL	17	15
Hall, 2014[34]	RL	15	15
Hegedus, 2018[35]	RL	46	59
Herzallah, 2013[36]	RL	22	13
Jollant, 2005[37]	RL	82	66
Jollant, 2016[38]	RL	30	24
Kumar, 2018[39]	RL	26	25
Liu, 2017[40]	RL	17	21
McGovern, 2014[41]	RL	36	60
Moutoussis, 2018[42]	RL	22	39
Must, 2006[43]	RL	20	30
Nord, 2018[44]	RL	28	26
Rothkirch, 2017[45]	RL	28	29
Saperia, 2019[46]	RL	51	43
Thoma, 2015[47]	RL	16	18
Walsh, 2018[48]	RL	42	46

OV=Option Valuation; RRV=Reward Response Vigour; RB=Reward Bias; RL=Reinforcement Learning; MDD=Depressed Group; HC=Healthy Control Group; U=Entirely Unmedicated Depressed Group; M=At Least Partially Medicated Depressed Group.

© 2020 American Medical Association. All rights reserved.

eMethods 3. Formulae used to convert study measures into Cohen's ds and associated variances[49]

The following formulae were used to convert study measures into Cohen's ds and associated variances:

$$d = \frac{M_1 - M_2}{SD_{pooled}}$$

Equation 1. Cohen's d from Means and Standard Deviations of 2 samples.
d is Cohen's d, M_1 is the mean of one sample M_2 is the mean of the other sample, SD_{pooled} is the pooled standard deviation of the two samples (please see below.)

$$SD_{pooled} = \sqrt{\frac{(N_1 - 1)SD_1^2 + (N_2 - 1)SD_2^2}{N_1 + N_2 - 2}}$$

Equation 2. Pooled standard deviation of 2 samples.
SD_{pooled} is the pooled standard deviation of the two samples, N_1 is the size of one sample N_2 is the size of the other sample, SD_1 is the standard deviation of one sample, SD_2 is the standard deviation of the other sample.

$$d = \frac{t}{\sqrt{\frac{1}{\frac{1}{N_1}+\frac{1}{N_2}}}}$$

Equation 3. Cohen's d from t-statistic.
d is Cohen's d, N_1 is the size of one sample N_2 is the size of the other sample, t is the t-statistic.

$$d = \sqrt{\frac{F}{\frac{1}{\frac{1}{N_1}+\frac{1}{N_2}}}}$$

Equation 4. Cohen's d from F-statistic.
d is Cohen's d, N_1 is the size of one sample N_2 is the size of the other sample, F is the F-statistic.

$$Var_d = \frac{N_1 + N_2}{N_1 \times N_2} + \frac{d^2}{2(N_1 + N_2)}$$

Equation 5. Variance on Cohen's d.
Var_d is the Variance on Cohen's d, N_1 is the size of one sample N_2 is the size of the other sample, d is Cohen's d.

© 2020 American Medical Association. All rights reserved.

eTable 6. The summary statistic(s) that were converted to a Cohen's d, Cohen's d and the Variance on 'd' for each included study.

Author, Year	t	F	M (HC)	SD (HC)	M (MDD)	SD (MDD)	d	Var_d
Baek, 2017[1]			0.289	0.199	0.339	0.301	0.191	0.024
Charpentier, 2017[2]			0.311	0.583	0.565	0.458	0.469	0.123
Chung, 2017[3]			0.500	0.310	0.460	0.310	0.129	0.057
Clarke, 2011[4]			0.920	0.725	0.672	0.876	0.294	0.060
Dombrovski, 2012[5]			0.608	0.608	0.624	0.671	-0.024	0.099
Murphy, 2001[6]			1.569	0.692	1.194	0.945	0.459	0.086
Subramaniapillai, 2019[7]			0.570	0.321	0.380	0.224	0.690	0.103
Treadway, 2012[8]			0.542	0.170	0.428	0.098	0.856	0.127
Yang, 2014[9]			0.682	0.286	0.580	0.323	0.334	0.042
Admon, 2017[10]			34.950	37.142	36.990	29.680	-0.061	0.098
Arrondo, 2015[11]			0.035	0.038	0.036	0.042	-0.025	0.089
Carl, 2016[12]		0.330					0.163	0.081
Chase, 2010[13]		5.637					-0.733	0.102
DelDonno, 2015[14]			-13.929	63.831	0.848	48.182	-0.259	0.099
DelDonno, 2019a[15]			18.159	23.440	4.347	13.432	0.678	0.187
DelDonno, 2019b[16]			5.359	15.226	5.489	14.828	-0.009	0.119
Pizzagalli, 2009[17]	2.090						0.535	0.068
Sankar, 2019[18]			7.310	21.650	4.270	26.460	0.126	0.100
Smoski, 2011[19]			23.880	23.170	28.310	28.040	-0.176	0.189
Takamura, 2017[20]			23.585	19.710	6.043	18.493	0.918	0.184
Xie, 2014[21]			5.610	15.810	4.175	11.483	0.110	0.075
Clery-Melin, 2011[22]			0.171	0.190	0.003	0.047	1.173	0.098
Aylward, 2019[23]			0.533	0.166	0.411	0.149	0.757	0.064
Henriques, 2000[24]	2.380						0.832	0.133
Lawlor, 2019[25]			0.106	0.170	0.102	0.208	0.021	0.032
Liu, 2011[26]			0.125	0.167	-0.068	0.128	1.298	0.056
Pizzagali, 2008[27]			0.228	0.171	0.101	0.184	0.716	0.089
Vrieze, 2013[28]	2.450						0.413	0.029
Alexopoulos, 2015[29]			0.07*	0.96	-0.04*	1.03	0.109	0.052
Cella, 2010[30]			4.510	8.810	0.230	8.587	0.492	0.106
Deisenhammer, 2018[31]			11.200	32.100	15.700	24.080	-0.166	0.059
Dezfouli, 2019[32]			0.635	0.110	0.575	0.116	0.534	0.061
Gradin, 2011[33]			50.590	1.800	48.500	1.190	1.352	0.154
Hall, 2014[34]		3.87					0.718	0.142
Hegedus, 2018[35]		4.171					0.402	0.039
Herzallah, 2013[36]			80.040	16.280	57.230	28.300	1.064	0.139
Jollant, 2005[37]			6.317	30.192	0.394	23.145	0.217	0.028
Jollant, 2016[38]			15.200	19.500	7.300	26.700	0.344	0.076

t=t-statistic; F=F-statistic; M=mean, SD=standard deviation; MDD=Depressed Group; HC=Healthy Control Group; d=Cohen's d; Var_d=Variance on d; *Z-scores.

© 2020 American Medical Association. All rights reserved.

eTable 6. The summary statistic(s) that were converted to a Cohen's d, Cohen's d and the Variance on 'd' for each included study (continued).

Author, Year	t	F	M (HC)	SD (HC)	M (MDD)	SD (MDD)	d	Var_d
Kumar, 2018[39]			87.820	12.510	80.370	19.180	0.462	0.081
Liu, 2017[40]			72.500	15.6	67.300	19.600	0.290	0.108
McGovern, 2014[41]			0.780	1.470	1.800	1.050	-0.834	0.048
Moutoussis, 2018[42]	0.277						0.074	0.071
Must, 2006[43]			6.400	4.510	-1.700	4.600	1.775	0.115
Nord, 2018[44]			75.100	15.124	64.120	17.888	0.665	0.078
Rothkirch, 2017[45]	1.010						-0.268	0.071
Saperia, 2019[46]			4.400	10.800	1.800	12.900	0.220	0.043
Thoma, 2015[47]			42.000	32.100	33.830	32.700	0.252	0.119
Walsh, 2018[48]	0.690						0.147	0.046

t=t-statistic; F=F-statistic; M=mean, SD=standard deviation; MDD=Depressed Group; HC=Healthy Control Group; d=Cohen's d; Var_d=Variance on d.

© 2020 American Medical Association. All rights reserved.

eResults. Moderator Analyses.
Of the total variation in effect sizes, 68% was due to between-study differences. The medication status of the MDD sample (unmedicated *vs* at least partially medicated) explained 0% of the variance in the global effect size (p=0.999). Additional models revealed no effect of medication status in any sub-component category. Those studies that clearly matched the case and control groups for gender (n=44) yielded a larger effect to those that didn't (p < 0.0001). This was influenced by one study where it was unclear whether they matched for gender[41] (SMD = -0.83 95% CI: -1.26 to –0.40). However, a significant difference remained between those studies that clearly balanced for gender (SMD = 0.389, 95% CI: 0.258 to 0.519, n=44) and those that didn't (SMD = 0.156, 95% CI: -0.289 to 0.599, n=3). Within the reward processing subcategories, gender-matching was only a moderator in the *Reinforcement Learning* subcategory (p<0.0002). As with the overall result, the one study in which it is unclear whether they balanced for gender[41] yielded the lowest effect size (please see above) while those studies that did not match for gender (n=2) yielded a lower summary effect SMD = 0.167, 95% CI: -0.397 to 0.732) than those that did (n=17, SMD = 0.444, 95% CI: 0.226 to 0.662). However, the proportion of female participants in the total study population did not have any effect on the reward processing effect size (p=0.840).

Studies that tested an exclusively elderly sample (n=4), yielded a smaller (p<0.001) and non-significant effect (SMD=-0.127, 95%CI: -0.555-0.300) than those that did not (n=44, SMD=0.390, 95%CI: 0.254-0.525). Again, within the reward processing subcategories, this was only significant for *Reinforcement Learning* (p < 0.0005) where those that tested elderly samples (n=2), yielded a lower (and insignificant: SMD=-0.367, 95% CI: -0.990 to 0.256) effect compared to the non-elderly samples (n=18) (SMD = 0.432, 95% CI: 0.209 to 0.654). In those studies that reported group anhedonia scores (n=17) or the result of a cold-cognitive task (n=8) we did not find any significant effects of either anhedonia or cold cognition on reward processing impairment (either overall, or in any subcategory). All other moderation analyses were non-significant.

© 2020 American Medical Association. All rights reserved.

Supplementary References

1. Baek K, Kwon J, Chae J-H, et al. Heightened aversion to risk and loss in depressed patients with a suicide attempt history. *Sci Rep*. 2017;7(1):11228. doi:10.1038/s41598-017-10541-5
2. Charpentier CJ, Aylward J, Roiser JP, Robinson OJ. Enhanced Risk Aversion, But Not Loss Aversion, in Unmedicated Pathological Anxiety. *Biol Psychiatry*. 2017;81(12):1014-1022. doi:10.1016/j.biopsych.2016.12.010
3. Chung D, Kadlec K, Aimone JA, McCurry K, King-Casas B, Chiu PH. Valuation in major depression is intact and stable in a non-learning environment. *Sci Rep*. 2017;7:44374. doi:10.1038/srep44374
4. Clark L, Dombrovski AY, Siegle GJ, et al. Impairment in risk-sensitive decision-making in older suicide attempters with depression. *Psychol Aging*. 2011;26(2):321-330. doi:10.1037/a0021646
5. Dombrovski AY, Siegle GJ, Szanto K, Clark L, Reynolds CF, Aizenstein H. The temptation of suicide: striatal gray matter, discounting of delayed rewards, and suicide attempts in late-life depression. *Psychol Med*. 2012;42(06):1203-1215. doi:10.1017/S0033291711002133
6. Murphy FC, Rubinsztein JS, Michael A, et al. Decision-making cognition in mania and depression. *Psychol Med*. 2001;31(4):679-693. http://www.ncbi.nlm.nih.gov/pubmed/11352370. Accessed July 31, 2018.
7. Subramaniapillai M, Mansur RB, Zuckerman H, et al. Association between cognitive function and performance on effort based decision making in patients with major depressive disorder treated with Vortioxetine. *Compr Psychiatry*. 2019;94. doi:10.1016/j.comppsych.2019.07.006
8. Treadway MT, Bossaller NA, Shelton RC, Zald DH. Effort-based decision-making in major depressive disorder: A translational model of motivational anhedonia. *J Abnorm Psychol*. 2012;121(3):553-558. doi:10.1037/a0028813
9. Yang X, Huang J, Zhu C, et al. Motivational deficits in effort-based decision making in individuals with subsyndromal depression, first-episode and remitted depression patients. *Psychiatry Res*. 2014;220(3):874-882. doi:10.1016/j.psychres.2014.08.056
10. Admon R, Kaiser RH, Dillon DG, et al. Dopaminergic Enhancement of Striatal Response to Reward in Major Depression. *Am J Psychiatry*. 2017;174(4):378-386. doi:10.1176/appi.ajp.2016.16010111
11. Arrondo G, Segarra N, Metastasio A, et al. Reduction in ventral striatal activity when anticipating a reward in depression and schizophrenia: a replicated cross-diagnostic finding. *Front Psychol*. 2015;6:1280. doi:10.3389/fpsyg.2015.01280
12. Carl H, Walsh E, Eisenlohr-Moul T, et al. Sustained anterior cingulate cortex activation during reward processing predicts response to psychotherapy in major depressive disorder. *J Affect Disord*. 2016;203:204-212. doi:10.1016/j.jad.2016.06.005
13. Chase HW, Michael A, Bullmore ET, Sahakian BJ, Robbins TW. Paradoxical enhancement of choice reaction time performance in patients with major depression. *J Psychopharmacol*. 2010;24(4):471-479. doi:10.1177/0269881109104883
14. DelDonno SR, Weldon AL, Crane NA, et al. Affective personality predictors of disrupted reward learning and pursuit in major depressive disorder. *Psychiatry Res*. 2015;230(1):56-64. doi:10.1016/j.psychres.2015.08.011
15. DelDonno SR, Mickey BJ, Pruitt PJ, et al. Influence of childhood adversity, approach motivation traits, and depression on individual differences in brain activation during reward anticipation. *Biol Psychol*. 2019;146. doi:10.1016/j.biopsycho.2019.05.009
16. DelDonno SR, Karstens AJ, Cerny B, et al. The Titrated Monetary Incentive Delay Task: Sensitivity, convergent and divergent validity, and neural correlates in an RDoC sample. *J Clin Exp Neuropsychol*. 2019;41(5):512-529. doi:10.1080/13803395.2019.1585519
17. Pizzagalli DA, Holmes AJ, Dillon DG, et al. Reduced Caudate and Nucleus Accumbens Response to Rewards in Unmedicated Individuals With Major Depressive Disorder. *Am J Psychiatry*. 2009;166(6):702-710. doi:10.1176/appi.ajp.2008.08081201
18. Sankar A, Yttredahl AA, Fourcade EW, et al. Dissociable neural responses to monetary and social gain and loss in women with major depressive disorder. *Front Behav Neurosci*. 2019;13. doi:10.3389/fnbeh.2019.00149
19. Smoski MJ, Rittenberg A, Dichter GS. Major depressive disorder is characterized by greater reward network activation to monetary than pleasant image rewards. *Psychiatry Res Neuroimaging*. 2011;194(3):263-270. doi:10.1016/j.pscychresns.2011.06.012
20. Takamura M, Okamoto Y, Okada G, et al. Patients with major depressive disorder exhibit reduced reward size coding in the striatum. *Prog Neuro-Psychopharmacology Biol Psychiatry*. 2017;79(Pt B):317-323. doi:10.1016/j.pnpbp.2017.07.006
21. Xie W, Li H, Luo X, et al. Anhedonia and Pain Avoidance in the Suicidal Mind: Behavioral Evidence for Motivational Manifestations of Suicidal Ideation in Patients With Major Depressive Disorder. *J Clin Psychol*. 2014;70(7):681-692. doi:10.1002/jclp.22055
22. Cléry-Melin M-L, Schmidt L, Lafargue G, Baup N, Fossati P, Pessiglione M. Why Don’t You Try Harder? An Investigation of Effort Production in Major Depression. Laks J, ed. *PLoS One*. 2011;6(8):e23178. doi:10.1371/journal.pone.0023178
23. Aylward J, Hales C, Robinson E, Robinson OJ. Translating a rodent measure of negative bias into humans: The impact of induced anxiety and unmedicated mood and anxiety disorders. *Psychol Med*. 2019.

© 2020 American Medical Association. All rights reserved.

doi:10.1017/S0033291718004117
24. Henriques JB, Davidson RJ. Decreased responsiveness to reward in depression. *Cogn Emot*. 2000;14(5):711-724. doi:10.1080/026999300050117684
25. Lawlor VM, Webb CA, Wiecki T V., et al. Dissecting the impact of depression on decision-making. *Psychol Med*. 2019. doi:10.1017/S0033291719001570
26. Liu W, Chan RCK, Wang L, et al. Deficits in sustaining reward responses in subsyndromal and syndromal major depression. *Prog Neuro-Psychopharmacology Biol Psychiatry*. 2011;35(4):1045-1052. doi:10.1016/j.pnpbp.2011.02.018
27. Pizzagalli DA, Iosifescu D, Hallett LA, Ratner KG, Fava M. Reduced hedonic capacity in major depressive disorder: evidence from a probabilistic reward task. *J Psychiatr Res*. 2008;43(1):76-87. doi:10.1016/j.jpsychires.2008.03.001
28. Vrieze E, Pizzagalli DA, Demyttenaere K, et al. Reduced Reward Learning Predicts Outcome in Major Depressive Disorder. *Biol Psychiatry*. 2013;73(7):639-645. doi:10.1016/j.biopsych.2012.10.014
29. Alexopoulos GS, Manning K, Kanellopoulos D, et al. Cognitive control, reward-related decision making and outcomes of late-life depression treated with an antidepressant. *Psychol Med*. 2015;45(14):3111-3120. doi:10.1017/S0033291715001075
30. Cella M, Dymond S, Cooper A. Impaired flexible decision-making in major depressive disorder. *J Affect Disord*. 2010;124(1-2):207-210. doi:10.1016/j.jad.2009.11.013
31. Deisenhammer EA, Schmid SK, Kemmler G, Moser B, Delazer M. Decision making under risk and under ambiguity in depressed suicide attempters, depressed non-attempters and healthy controls. *J Affect Disord*. 2018;226:261-266. doi:10.1016/j.jad.2017.10.012
32. Dezfouli A, Griffiths K, Ramos F, Dayan P, Balleine BW. Models that learn how humans learn: The case of decision-making and its disorders. *PLoS Comput Biol*. 2019;15(6). doi:10.1371/journal.pcbi.1006903
33. Gradin VB, Kumar P, Waiter G, et al. Expected value and prediction error abnormalities in depression and schizophrenia. *Brain*. 2011;134(6):1751-1764. doi:10.1093/brain/awr059
34. Hall GBC, Milne AMB, MacQueen GM. An fMRI study of reward circuitry in patients with minimal or extensive history of major depression. *Eur Arch Psychiatry Clin Neurosci*. 2014;264(3):187-198. doi:10.1007/s00406-013-0437-9
35. Hegedűs KM, Szkaliczki A, Gál BI, Andó B, Janka Z, Álmos PZ. Decision-making performance of depressed patients within 72 h following a suicide attempt. *J Affect Disord*. 2018;235:583-588. doi:10.1016/j.jad.2018.04.082
36. Herzallah MM, Moustafa AA, Natsheh JY, et al. Learning from negative feedback in patients with major depressive disorder is attenuated by SSRI antidepressants. *Front Integr Neurosci*. 2013;7:67. doi:10.3389/fnint.2013.00067
37. Jollant F, Bellivier F, Leboyer M, et al. Impaired Decision Making in Suicide Attempters. *Am J Psychiatry*. 2005;162(2):304-310. doi:10.1176/appi.ajp.162.2.304
38. Jollant F, Richard-Devantoy S, Ding Y, Turecki G, Bechara A, Near J. Prefrontal inositol levels and implicit decision-making in healthy individuals and depressed patients. *Eur Neuropsychopharmacol*. 2016;26(8):1255-1263. doi:10.1016/j.euroneuro.2016.06.005
39. Kumar P, Goer F, Murray L, et al. Impaired reward prediction error encoding and striatal-midbrain connectivity in depression. *Neuropsychopharmacology*. 2018;43(7):1581-1588. doi:10.1038/s41386-018-0032-x
40. Liu W-H, Valton V, Wang L-Z, Zhu Y-H, Roiser JP. Association between habenula dysfunction and motivational symptoms in unmedicated major depressive disorder. *Soc Cogn Affect Neurosci*. 2017;12(9):1520-1533. doi:10.1093/scan/nsx074
41. McGovern AR, Alexopoulos GS, Yuen GS, Morimoto SS, Gunning-Dixon FM. Reward-related decision making in older adults: relationship to clinical presentation of depression. *Int J Geriatr Psychiatry*. 2014;29(11):1125-1131. doi:10.1002/gps.4200
42. Moutoussis M, Rutledge RB, Prabhu G, et al. Neural activity and fundamental learning, motivated by monetary loss and reward, are intact in mild to moderate major depressive disorder. *PLoS One*. 2018;13(8). doi:10.1371/journal.pone.0201451
43. Must A, Szabó Z, Bódi N, Szász A, Janka Z, Kéri S. Sensitivity to reward and punishment and the prefrontal cortex in major depression. *J Affect Disord*. 2006;90(2-3):209-215. doi:10.1016/j.jad.2005.12.005
44. Nord CL, Lawson RP, Huys QJM, Pilling S, Roiser JP. Depression is associated with enhanced aversive Pavlovian control over instrumental behaviour. *Sci Rep*. 2018;8(1). doi:10.1038/s41598-018-30828-5
45. Rothkirch M, Tonn J, Köhler S, Sterzer P. Neural mechanisms of reinforcement learning in unmedicated patients with major depressive disorder. *Brain*. 2017;140(4):1147-1157. doi:10.1093/brain/awx025
46. Saperia S, Da Silva S, Siddiqui I, et al. Reward-driven decision-making impairments in schizophrenia. *Schizophr Res*. 2019;206:277-283. doi:10.1016/j.schres.2018.11.004
47. Thoma P, Norra C, Juckel G, Suchan B, Bellebaum C. Performance monitoring and empathy during active and observational learning in patients with major depression. *Biol Psychol*. 2015;109:222-231. doi:10.1016/J.BIOPSYCHO.2015.06.002
48. Walsh AEL, Browning M, Drevets WC, Furey M, Harmer CJ. Dissociable temporal effects of bupropion on behavioural measures of emotional and reward processing in depression. *Philos Trans R Soc B Biol Sci*. 2018;373(1742). doi:10.1098/rstb.2017.0030

© 2020 American Medical Association. All rights reserved.

49. Lakens D. Calculating and reporting effect sizes to facilitate cumulative science: a practical primer for t-tests and ANOVAs. *Front Psychol*. 2013;4(NOV):863. doi:10.3389/fpsyg.2013.00863

© 2020 American Medical Association. All rights reserved.
